# Supplementary material for: Evaluation of Thermal Changes of the Sole Surface in Horses with Palmar Foot Pain: A Pilot Study
Source: Biology (Basel). 2023 Mar 10;12(3):423. doi: 10.3390/biology12030423 (PMC10045226; doi:10.3390/biology12030423)
Supplement: Supplementary file 1 [file biology-12-00423-s001.zip › Tables S1 - S6 Thermography results – Temperature for toe (El2) and frog (El1) areas.pdf]

Thermography results – Temperature for toe area El2

Table S1: Study group horses with palmar foot pain: Temperature of the toe area (El2)

Specimen	Minimum temperature before/after training, °C	Mean temperature before/after training, °C
1	20.5 / 27.4	22.8 / 31.7
2	19.5 / 28.9	23.0 / 31.4
3	21.1 / 28.8	24.4 / 29.8
4	21.4 / 28.6	25.2 / 29.3
5	20.3 / 28.9	24.6 / 31.1
6	20.2 / 27.9	23.7 / 29.1
7	21.1 / 27.5	23.7 / 29.5
8	20.7 / 24.2	24.1 / 28.7

Table S2: Study group non-lame limb: Temperatures of the toe area (El2)

Specimen	Minimum temperature before/after training, °C	Mean temperature before/after training, °C
1	27.8 / 29.3	27.3 / 29.9
2	26.9 / 28.2	28.3 / 29.6
3	27.8 / 29.1	28.4 / 30.0
4	28.5 / 29.0	29.3 / 30.1
5	27.3 / 28.6	28.3 / 29.4
6	28.2 / 29.6	28.4 / 29.7
7	27.5 / 28.3	28.2 / 28.9
8	27.6 / 29.0	29.3 / 30.2

Table S3: Control group: Temperatures of the toe area (El2)

Specimen	Minimum temperature before/after training, °C	Mean temperature before/after training, °C
1	24.7 / 26.1	26.7 / 27.5
2	23.9 / 25.1	26.3 / 28.0
3	22.5 / 25.7	25.6 / 27.2
4	25.1 / 27.4	27.2 / 27.9

Thermography results – Temperature for frog area El1

Table S4: Study group horses with palmar foot pain: temperature of the frog area (El1)

Specimen	Minimum temperature before/after training, °C	Mean temperature before/after training, °C
1	19.8 / 21.1	20.9 / 23.8
2	21.2 / 22.1	22.6 / 24.1
3	20.8 / 21.5	22.2 / 23.5
4	19.9 / 20.0	21.3 / 21.7
5	21.3 / 23.1	22.9 / 24.0
6	20.7 / 22.3	21.9 / 26.2
7	21.1 / 22.6	23.0 / 25.1
8	23.6 / 24.8	24.6 / 26.0

Table S5: Study group non-lame limb: Temperatures of the frog area (El1)

Specimen	Minimum temperature before/after training, °C	Mean temperature before/after training, °C
1	26.4 /28.6	27.9 / 29.3
2	25.5 / 29.2	29.9 / 29.7
3	26.5 / 27.8	27.4 /28.8
4	28.5 / 30.8	29.9 / 31.9
5	26.2 / 28.4	28.2 / 29.4
6	27.2 / 28.3	28.3 / 30.1
7	26.9 / 28.7	27.7 / 29.1
8	26.7 / 28.4	27.3 / 29.3

Table S6: Control group: Temperatures of the frog area (El1)

Specimen	Minimum temperature before/after training, °C	Mean temperature before/after training, °C
1	22.3 / 25.6	24.1 / 26.8
2	23.9 / 24.7	25.5 / 26.1
3	23.0 / 25.8	24.3 / 27.4
4	22.3 / 24.7	25.1 / 26.1
