# Supplementary figures and images for: Evaluation of Thermal Changes of the Sole Surface in Horses with Palmar Foot Pain: A Pilot Study
Source: Biology (Basel). 2023 Mar 10;12(3):423. doi: 10.3390/biology12030423 (PMC10045226; doi:10.3390/biology12030423)

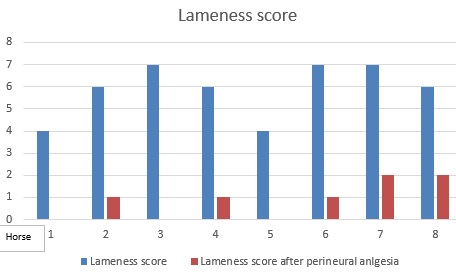

Supplement: Supplementary file 1 [file biology-12-00423-s001.zip › Figure S1- lameness score.jpg]

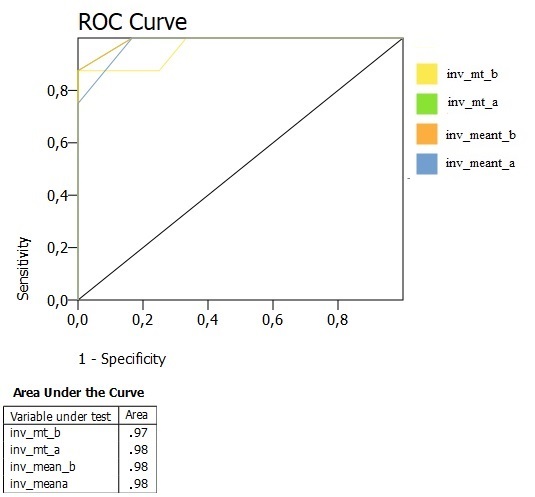

Supplement: Supplementary file 1 [file biology-12-00423-s001.zip › Figure S2 - ROC curve frog area.jpg]

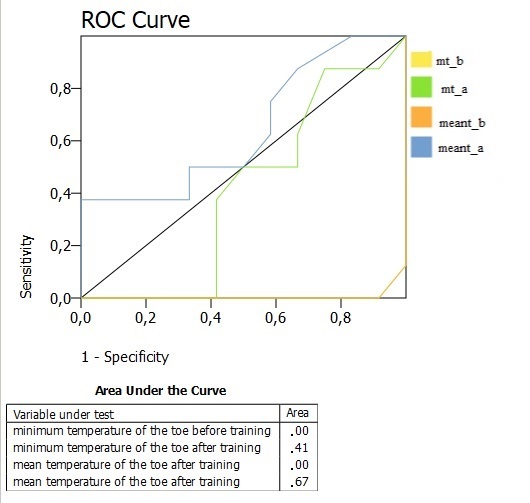

Supplement: Supplementary file 1 [file biology-12-00423-s001.zip › Figure S3 - ROC curve toe area.jpg]
